# Ethical challenges of caring for burn patients: a qualitative study

**DOI:** 10.1186/s12910-021-00582-x

**Published:** 2021-02-10

**Authors:** Mostafa Bijani, Fateme Mohammadi

**Affiliations:** 1 Department of Medical Surgical Nursing, Fasa University of Medical Sciences, Fasa, Iran; 2 Chronic Diseases (Home Care) Research Center, Autism Spectrum Disorders Research Center, Department of Nursing, School of Nursing and Midwifery, Hamadan University of Medical Sciences, Hamadan, Iran

## Abstract

**Background:**

Burn patients are among the most vulnerable groups of patients requiring principled ethical care. Caring for these patients often brings various ethical challenges for the members of the health care teams, especially nurses, which affect the clinical decisions made for these patients. A limited number of studies have addressed the ethical challenges of caring for burn patients for the responsible caregivers, so the present study attempted to identify these challenges. The present study aimed to explore the health professionals' experiences of the ethical challenges during caring for burn patients.

**Methods:**

This was a qualitative study with a descriptive, phenomenological design. 22 health professional practiced in public burn centers in Iran who met the inclusion criteria of the study were selected via purposeful sampling to participate in the study from June to August 2019. Data were collected using semi-structured, in-depth interviews with the individuals as well as field notes. Sampling was continued up to the data saturation. Thereafter, the collected data were analyzed using Colaizzi's method.

**Results:**

The findings of the study yielded 3 themes, including respect for the patient's privacy, respect for the patient's personal identity, and care challenges, as well as 9 categories.

**Conclusion:**

The findings of this study showed that the burn patients’ caregivers face some challenges in the domains of maintaining the patient's privacy, respecting the patient’s personal identity, and making the best clinical decision. Thus, providing the cultural, professional, and organizational requirements of meeting the challenges of caring for burn patients can consequently result in the caregivers’ inner peace and the improved performance.

## Background

Burns and their consequent damages are among the most serious healthcare issues, which are considered as the main causes of fatality and disability worldwide [1]. According to official statistics in Iran, 30,000 burn patients are annually admitted in hospitals, of them about 3000 subjects die due to the severity of their burns [2]. Burns rank as the sixth on the list of top known causes of death in Iran [2, 3]. Additionally, in Iran, common causes of burns in patients are hot liquids and objects, explosions, chemicals, electric shocks, and self-immolation [4]. Although self-immolation is low in developed countries, self-immolation rates are high in developing countries such as Iran [5]. Moreover, in Iran, 39.1 to 40% of suicide attempts have been reported through self-immolation, which is more observed in women than men [6, 7]***.*** Therefore, the burns unit is one of the most stressful and challenging parts of health care centers, because burn patients suffer from various problems, including pain, frequent bandaging, and fear of deformation of the body parts that inevitably affect the patients’ daily and physical activities, social, psychological and professional status, and quality of life [8]. Therefore, the most important responsibilities of the health care members are paying attention to the burn patients’ nutritional needs, pain relief, control and prevention of wound infection, physiotherapy, and medication [9]. In this regard, nurses constitute the largest part of health care teams who are responsible for round-the-clock care and treatment of patients [10–13]. Several studies indicated that health professions experience the majority of workload and physical and mental stress in caring for burn patients, because of their responsibilities for controlling and relieving their pain, witnessing deformities caused by burns, considering the patients' emotional and psychological needs, and observing ethical principles in care that are among the most significant challenges the nurses have to deal with [11, 14]. These studies reported that health professionals of burn patients encounter complex clinical and ethical challenges, including the manner of allocating resources, justice in treatment, lack of harm to the patient in the process of treatment and care, respect for the patient's autonomy, issues related to skin transplant, and consideration of the patient’s preferences in choosing, and accepting a treatment modality, thus warranting immediate investigations and interventions [15, 16]. Despite many attempts to investigate the challenges of caring for burn patients, the ethical challenges of this health care area have not been identified and explored in detail yet. Health professionals of burns units spend long times with burn patients, so they are considered as their close companions who understand them quite well. Accordingly, they were selected as the most reliable sources for identifying and investigating the ethical challenges during caring for burn patients. Given the significance of studying the ethical challenges of caring for burn patients in Iran, the present study mainly addressed the caregivers’ experiences of the ethical challenges during caring for burn patients. Hopefully, the findings of the study would enable the administrators and caregivers to provide a supportive environment where burn patients' rights are properly respected. Therefore, the present study aimed to provide a description of the health professionals' experiences of the ethical challenges of caring for burn patients.

## Method

This study was a qualitative research with a descriptive phenomenology approach. The purpose of this study was exploring ethical challenges in caring of burn patients from the perspective of their professional caregivers. Therefore, 22 health professionals were selected from three burn wards in governmental hospitals affiliated with a University of Medical Sciences in southeastern Iran using purposeful sampling method who were then enrolled in the study from June to August 2019. Notably, purposeful sampling is a method used in qualitative research to identify and select the individuals or groups of individuals who are especially knowledgeable about or experienced in a phenomenon. In this method of sampling, besides knowledge and experience, one should consider the importance of availability and willingness to participate, as well as the ability to communicate experiences and opinions in an articulate, expressive, and reflective manner [17].

Accordingly, the inclusion criteria were having at least two years of work experience in providing care for burn patients, being Iranian, speaking and understanding Persian language, and being able to provide appropriate and sufficient information on the subject. There was an attempt to select the participants of this study with maximum variation. Therefore, health professionals were selected from a wide range of age, sex, marital status, and educational levels. In this study, 26 nurses, 4 dermatologists, and one nutritionist who were working in 3 burn care units, were included. Although all of them were invited to participate in this study, 20 nurses and 2 dermatologists agreed to be enrolled. The participants’ information are shown in Table 2.

In this study, data generation was done through individual and semi-structural interviews and field notes. These interviews were conducted face-to-face in a quiet environment when the participants were assigned. The corresponding author began individual interviews using universal questions and then guided them by asking the following original questions: “Explain your experiences regarding ethical challenges during caring of burn patients’’ ‘“What do ethical challenges in caring of burn patients’ mean to you?”, and “Which factors do you think can help in decreasing ethical challenges during caring of burn patients?” According to the participants' answers to these questions, some follow-up questions such as “Please explain more?” or “Can you give an example?” were then asked. Each interview lasted about 55–90 min. The interviews were recorded after obtaining the written consent from all the participants. Immediately after performing each interview, the interviews were listened by the corresponding author several times, in order to develop a general understanding and deep insights. Subsequently, they were transcribed on paper, along with the field notes, and then organized manually. After each interview, the data were analyzed and the next interview was scheduled. The interviews continued until no other new sub-themes were created and data reached the segregation.

Data analysis was done based on the Colaizzi’s seven-step method as follows: Step one: Familiarization (each interview was listened and then transcribed, and each transcript was studied several times to gain a sense of the whole content). Step two: Identification of significant statements (statements were extracted). Step three: Formulation of meanings (meanings were formulated from the significant statements). Step four: Formation of cluster of themes (all the formulated meanings were allocated to subthemes with a unique structure). Step five: Development of an exhaustive description (all the emergent themes were provided with an exhaustive description and the findings were then integrated into a comprehensive description of the phenomenon). Step six: Production of the fundamental structure (a comprehensive description of the phenomenon studied in the form of an explicit statement set, was formulated). Step seven: Search for the verification of the fundamental structure (the results were returned to the participants to be confirmed and validated) [13]. An example of the analysis is shown in Table 1.

**Table 1:** An example of an analysis

Step 1	Step 2	Step 3	Step 4	Step 5	Step 6	Step 7
Interview was recorded and transcribed	Some patients have no hope for surviving and living a quality life. They continuously ask their treatment team, especially the nurses with whom they have the most interaction, to stop trying to save them and let them die. They desire death	Abandoning hope for recovery and survival requesting palliative care as well as an easy death desiring death	Peaceful death	Euthanasia	Challenges of decision-making	Member checking was done To do this, the extracted concepts and themes were submitted to 4 participants who confirmed that the findings are in line with their understandings and interpretations

Thereafter, Guba and Lincoln criteria were used to evaluate the trustworthiness. These criteria include credibility (confidence in the 'truth' of the findings), dependability (showing that the findings are consistent, so they could be repeated), transferability (showing that the findings have applicability in other contexts), and conformability (a degree of neutrality or the extent to which the findings of a study are shaped by respondents and not by researcher bias, motivation or interest) [17]. For this purpose and in order to increase the acceptability and accuracy of the obtained data, we performed data generation using two techniques of semi-structured interviews and field notes, and prolonged engagement and drowning in data. Moreover, the researcher restricted the textual reviews in order to reduce bias in collecting, analyzing, and coding member checking. The extracted concepts and themes were submitted to 4 participants and 3 peers who stated that these findings are in line with their understandings and interpretations; therefore, both member and peer checking were done.

## Results

In the present study, 22 caregivers (13 women and 9 men) within an age range of 26–46 years old from 3 public burns centers were interviewed. The majority of the participants were married (60%) and had a bachelor's degree in nursing (65%) with at least 2 years of work experience. The personal characteristics of the participants are shown in Table 2. Three main themes (including respecting the patient's privacy, respecting the patient's personal identity, and making the best clinical decision) as well as nine categories were extracted from the collected data (Table 3).

**Table 2:** Individual social characteristics of the participants

Participants	Sex	Marital status	Educational level	Work experience (years)
P1	Female	Single	Bachelor's degree in nursing	13
P2	Female	Married	Bachelor's degree in nursing	10
P3	Male	Married	Bachelor's degree in nursing	18
P4	Female	Married	Bachelor's degree in nursing	2
P5	Male	Married	Associate's degree in Nursing	8
P6	Female	Single	Associate's degree in Nursing	7
P7	Female	Married	Associate's degree in Nursing	9
P8	Male	Married	master's degree in nursing	10
P9	Female	Single	master's degree in nursing	8
P10	Male	Married	Bachelor's degree in nursing	18
P11	Female	Married	Bachelor's degree in nursing	5
P12	Female	Single	Associate's degree in nursing	15
P13	Male	Single	Bachelor's degree in nursing	3
P14	Male	Married	Bachelor's degree in nursing	2
P15	Female	Married	Bachelor's degree in nursing	2
P16	Female	Single	Bachelor's degree in nursing	13
P17	Female	Married	Associate's degree in Nursing	5
P18	Male	Married	Bachelor's degree in nursing	3
P19	Female	Single	Bachelor's degree in nursing	7
P20	Male	Single	Bachelor's degree in nursing	15
P20	Female	Single	Specialist skin Diseases	8
P20	Male	Married	Specialist skin Diseases	9

**Table 3:** Themes and categories extracted from the descriptive phenomenological approach

Theme	Category	Quotations
Respect for privacy	Respect for physical-sexual privacy	"Bandaging burns is very time-consuming and can take a long time. After baths, the patients' bodies are in full view of the person who is in charge of bandaging. Even when they are visited by a doctor who is from the opposite sex, the patients feel uncomfortable, especially if the patient is woman and the burn has affected her genitalia or a contiguous area. They feel embarrassed and say that if their caregiver and doctor are of the same gender as them, they will feel more secure" (Participant 3)
	Respect for psychological privacy	"Some of these patients suffer from personality disorders, so they have tried self-immolation. When they are educating these patients, the caregivers disregard their conditions and personality disorders—if a patient becomes aggressive, they call them with inappropriate names, like crazy, psycho, etc. and deteriorate their psychological status" (Participant 7)
	Maintaining confidentiality	"Keeping patients' secrets is a basic ethical principle, but it is especially essential in the burns center. Burn patients are more likely to have a history of psychological issues, personality disorders, childhood abuse or various family problems; since in our country, there is a very negative attitude toward such matters, it is important that all their information should remain confidential. The patients do not want anyone to know about their past, except their main caregiver and doctor. Occasionally, medical records are read by non-medical staff, so it is better that ward administrators keep these information confidential" (Participant 9)
Respect for personal identity	Respect for personal identity	"At times, some families bring sheets of green cloth and ask us to spread them on their patients with extensive burn. Well, such a thing will spread infection and is not safe for the patient. We try to explain this to these families, but they do not listen and say it is a part of their beliefs, which can heal their patient. No matter how hard we try, they refuse to listen. Well, sometimes nurses who are under pressure at work may lose their temper and say things that are considered as offensive to the patients and their families. In short, we have learned how to stop them from putting the cloth on their patients by persuading them to just put it beside them; using this way, we maintain the patients' dignity as best as we can" (Participant 12)
	Avoidance of pitying behaviors	"It is not right for nurses to take uncalled for pity on such patients and cast pitying looks at them. Pitying behaviors undermine the patients' self-esteem and can make them feel disinclined to cooperate with the nurses or even demonstrate aggressive behaviors" (Participant 20)
	Avoidance of discrimination	"Occasionally, the medical team and the nurses differentiate between self-immolation survivors and those whose burns are caused by an accident, and they care for them and treat them differently. They do not communicate much with the patients who have tried self-immolation and let the ward counselors and psychologists take care of them. They have more extensive relationships with the patients who are victims of fire accidents and treat them with more compassion. These kinds of behaviors are disturbing to the patients" (Participant 14)
Clinical challenges	Lack of time and loss of hope	"The nurses get to spend longer hours with the patients, which inevitably makes the patients feel more comfortable with them compared to the other members of their treatment team; they prefer to talk to and have more contact with their nurses. The problem is that workload in the burns center is high, bandaging and administration of medications can take hours, and the nurse end up with little time for having any clinical interaction with the patients. This can frustrate the patients and diminish their interactions with the healthcare team. Additionally, this is a serious issue in providing ethical and methodical care to burn patients" (Participant 18)
	Defective teamwork	"Sometimes, there is not enough coordination between the activities of the treatment team. For example, the psychologist or physiotherapist start their rounds when it is time for a patient's bandaging or medication and insist that they should communicate with the patient or they intend to interact with a patient who has just had a painful dressing. Well, at such times, patients are willing to interact with no one. Patients are more willing to interact in the evening and at night, but those are the times when there is no resident psychologist in the hospital. This lack of coordination in teamwork disrupts caregiving (Participant 1)
	Euthanasia	"The patients with severe burns are in great pain and have no hope for survival or living a high-quality life again. Some of them keep asking their caregivers, especially the nurses who have the most interaction with them, to stop trying to save them and prolonging their agony. Passive euthanasia is not legal in our country and the treatment team is challenged by how to respond to such requests" (Participant 16)

### Respecting the patient's privacy

The participants reported that respecting the burn patients' physical-sexual and psychological privacies and maintaining their confidentiality, which are essential to maintain their dignity, are among the most important challenges faced by the caregivers. Caregivers are expected to respect their patients' privacy. The theme of respecting the patient's privacy consists of the following three categories: respecting the patient's physical-sexual privacy, respecting the patient's psychological privacy, and maintaining confidentiality.

#### Respecting the patient's physical-sexual privacy

The participants stated that hospitalized burn patients, most of whom have committed self-immolation, are suffering from various physical and mental issues, and caring for them is very demanding. In some cases, a large part of the patient's body, including the genitalia, is affected. It is recommended that such patients should be cared by caregivers of the same gender.

#### Respecting the patient's psychological privacy

Some of the participating caregivers mentioned the significance of respecting the burn patients' psychological privacy. In the case of patients who have committed self-immolation or have a history of any psychological disorder, it is essential that caregivers respect their psychological privacy, consider their psychological status while providing care and education to them, and not remind them their shortcomings in order to prevent ruining their self-esteem.

#### Maintaining confidentiality

The participants stated that the burn patients' personal information and especially their history of psychological disorders and childhood abuse, if any, must not be mentioned in their medical records, and should be treated as confidential by head nurses and doctors.

### Respecting the patient's personal identity

In the present study, the participating caregivers pointed out that burn patients need to be respected regarding their religious identity, values, and beliefs. Additionally, toward maintaining the dignity of these patients, caregivers should avoid pitying and discriminatory behaviors, but they should fairly provide them with treatment. This theme consists of the following three categories: respecting the patient's religious identity, avoiding pitying behaviors, and avoiding discrimination.

#### Respecting the patient's religious identity

Respecting the patients' religious identity, values, and beliefs is one of the ethical principles of nursing, which can guarantee justice in care if performed appropriately. The participants noted that some burn patients and their families have certain religious-spiritual beliefs and values, which help them coping with their painful and difficult situation. However, since the performance of certain religious rituals can increase the risk of infection in burns centers, nurses must respect the patients' beliefs and values as long as the patients' safety is not threatened.

#### Avoiding from pitying behaviors

The participants mentioned that caregivers must act professionally toward burn patients and avoid pitying behaviors and looks.

#### Avoiding discrimination

Moreover, caregivers must not discriminate against burn patients with psychological disorders and those who have committed self-immolation. In addition, the caregivers are required to treat these patients and those whose burns are due to fire accidents equally and provide care for them fairly.

### Clinical challenges

The participants stated work overload and the consequent defects in provision of quality care, shortage of time, and poor coordination in teamwork among the main ethical challenges of caring for burn patients. This theme consists of the following three categories: shortage of time and frustration, defective teamwork, and euthanasia.

#### Shortage of time and frustration

According to the participants, caring for burn patients is highly demanding and time-consuming. In this regard, each bandaging takes hours to be completed, there is a huge workload, and the number of nurses in each shift is inadequate. The nurses cannot afford the time to sympathize and interact with the patients, the fact that is considered as a major barrier to provide quality care.

#### Defective teamwork

The caregivers remarked that, occasionally, the interactions among the members of the treatment team were not satisfactory and poor coordination consequently resulted in physical and psychological tensions to patients. Correspondingly, this is against one of the most important ethical principles in healthcare, namely protecting the patient from injuries and tension.

#### Euthanasia

The participating nurses in this study referred to euthanasia and death as the factors among the most difficult ethical-clinical challenges of caring for burn patients. The endless moaning and suffering of these patients, some of whom have no hope for surviving or living a normal life another time, and their repeated requests for passive euthanasia present a dreadful ethical challenge to nurses and the other members of the healthcare team.

## Discussion

Caring for burn patients is accompanied with various ethical and clinical challenges [11, 14, 16]. Since burn patients are more vulnerable than other patients, caring for them involves ethical challenges [14, 15]. Providing quality care based on professional codes of ethics is the main ethical and professional responsibility of caregivers of burn patients suffering from physical and psychological disabilities. The present study was conducted to identify the ethical challenges of caring for burn patients from the nurses' perspective. In this regard, three main themes were extracted from the collected data as follows: respecting the patient's privacy, respecting the patient's personal identity, and clinical challenges. These findings are obtained from Iranian culture and society, so based on the generalizability of qualitative research, not all findings can be generalized to other cultures and societies. The ethical challenges of caring for burn patients have not been explored by other researchers in other studies. Therefore, due to a lack of relevant studies, the findings of the present study can be compared with the findings of other studies on the challenges of caring either for burn patients or for those with some other diseases. Maintaining their dignity in clinical settings is one of the patients' basic rights, which is also known as a major ethical challenge with that nurses have to deal with in practice. When providing care, nurses are required to respect the patients' privacy [18].

In the present study, respecting patients' privacy was found to have three categories as follows: respecting the patient's physical-sexual privacy, respecting the patient's psychological privacy, and maintaining confidentiality. Recent studies in Iran have mainly focused on maintaining the patients' dignity in clinical environments in terms of respecting their physical and psychological privacies and maintaining their confidentiality and regard it as a highly influential factor on promoting ethical and methodical care [3]. Correspondingly, this indicates the significance of respecting privacy in deferent cultures, especially in Iranian society. However, these studies reported that, occasionally, due to work overload, fatigue due to work in consecutive shifts, and shortage of personnel in wards, nurses fail to provide appropriate ethical care characterized by maintaining the patients' dignity [3, 19, 20]. On the other hand, due to the Iranian culture, and their religious beliefs and values, people are so sensitive about keeping their bodies covered, especially their genitalia, and Muslim men and women should avoid any physical contact with opposite sex. Accordingly, it is recommended that burn patients receive care from caregivers of the same gender, so in this situation, the patients' dignity is maintained. Respecting burn patients' psychological privacy was another important category referred by the caregivers. Several studies reported that respecting the psychological privacy of inpatients, especially burn patients, is essential in ethical care [21]. On the other hand, people in Iran face lots of stresses and psychological challenges due to social, economic, and cultural problems and they sometimes commit self-immolation to get rid of these conditions or do it as a sign of protest [5–7]. Therefore, many burn patients are self-immolation survivors suffering from psychological and personality disorders and occasionally have no control over their thoughts and behaviors. These patients are highly nervous, aggressive, and sensitive, so they may react to their treatment team and caregivers with aggression. Caregivers must be sympathetic to the patients under these conditions and must not address them angrily or stigmatize them, but they should treat them with kindness so that their dignity is not undermined. However, sometimes, in the face of their patients' disrespectful behaviors, nurses and other caregivers experience ethical distress, which is one of the major ethical challenges during caring for burn patients.

The caregivers also mentioned that it is important for the patients and their families that the nurses keep their personal information, medical status, psychological disorders, and personal life issues confidential and also inaccessible to non-clinical individuals and other patients. In Iran, besides the health care team (therapists, nutritionists, and physiotherapists), sometimes non-clinicians, including the ward’s secretary, cleaning workers, and administrative staff are present in the ward. They hear what the medical staff are saying about the patient and sometimes tell the patient’s information to other people subconsciously. Consequently, this upsets the patient and his/her family. Therefore, it is necessary for the medical staff to not present confidential patient information in the corridor, station or patients' beds. They should also talk about each patient together in a completely private environment, not in the ward’s conference room. Similarly, several studies mentioned maintaining the patients’ confidentiality as an important aspect of respecting their privacy, which is consistent with the findings of the present study [22–24].

Respecting the patient's personal identity was another theme extracted from the findings of this study. Respecting the patients' beliefs, values, and religious differences and avoiding from pitying behaviors and discrimination toward providing just care are among the major ethical challenges that should be considered in burns centers. In the present study, the theme of respecting the patient's personal identity include respecting religious identity, avoidance of pitying behaviors, and avoidance of discrimination. Likewise, other similar studies refer to respecting the patients' personal identities, while providing care to them, as an ethical challenge in healthcare [25–27]. In the present study, the caregivers remarked that respecting the religious beliefs and values of patients and their families is one of the main ethical challenges in the burns center. Sometimes, the beliefs and values of the patients and their families interfere with the principles of care and infection control. Many studies emphasized the importance of respecting the patients' beliefs and values [26, 27]. The participants also stated that it was important for them to not see pitying looks and behaviors from their caregivers along with excessive and non-clinical expressions of sympathy while caring for burn patients, because undue sympathy and pity-taking make them nervous and aggressive and undermine their self-esteem. Other studies indicated that sympathizing with patients, especially severely ill and terminal patients, creates positive feelings in patients, but pitying has a destructive effect on the patients' psychological health and consequently on self-esteem [26, 28]. Avoiding discrimination is another important category stated under the theme of respecting the patient's personal identity. The participants mentioned that caregivers should not differentiate between burn patients in terms of the cause of burn, background disease or personal beliefs. Discriminatory behavior by caregivers disrupts the process of providing professional care and is known as a major challenge in caring for burn patients. Avoidance of discrimination is a professional value in nursing, so caregivers are expected to avoid differentiating between patients [28]. Similarly, other studies provided evidence on that caregivers discriminate certain patients and ignore the above-mentioned ethical value [28, 29, 30].

The final theme extracted from the collected data was the challenges of making clinical decisions in caring for burn patients. The caregivers stated that, due to their heavy workload, treatment teams’ members and especially nurses do not have enough time for interacting and socializing with patients. Accordingly, this frustrates the patients about communicating with their caregivers, which is a way for patients to alleviate their emotional and psychological tensions. In addition, work overload and shortage of medical personnel result in incoordination during team working. Similarly, Sorlie et al. reported that shortage of time and patients' loss of hope and disruptions in teamwork are among serious ethical challenges in intensive care units, which are often caused by work overload and lack of time for providing methodical care [31]. Euthanasia is another important category related to the ethical challenges of making clinical decisions for burn patients. The caregivers stated that sometimes the extent of burn and injury is so much that patients prefer rather to die. Endless pain, an altered mental image of one's body, and loss of hope for recovery and living a normal life again lead some burn patients to ask from their treatment teams to stop the treatment and caring for them and let them die. In Iran, euthanasia, both active and passive, is illegal, as Muslims believe that life is a gift from God and caregivers are expected to not only refrain from doing any harm to their patients, but also they must make every effort to save their lives. Thus, patients' and their families' requests cannot stop the provision of clinical care, and this fronts caregivers with the greatest ethical challenge in their clinical decision-making for burn patients. Many other studies referred to euthanasia as a major ethical challenge in the clinical decisions, which must be made by caregivers for critically ill patients or those suffering from a debilitating disease [32–34].

Finally, the ethical challenges of caring for burn patients are among the most serious issues faced by treatment teams. The participants of the present study believed that caregivers should provide ethical and methodical care in an atmosphere where the patients' dignity and personal identity are respected.

## Limitation

The limitation of this study was the fact that the data were only collected via face-to-face interviews and field notes. Other methods of data collection can enrich the findings of a similar qualitative work of research. So, it is suggested that future studies, in addition to face-to-face interviews, should rely on some other methods of collecting qualitative data, including field observation and focus groups.

## Conclusion

Burn patients are among the most vulnerable individuals and caring for them is accompanied with various ethical challenges for the treatment team, especially nurses. Thus, there is a crucial need for identifying and understanding the ethical challenges of caring for burn patients. The findings of the present study showed that respecting the patient's privacy, respecting the patient's personal identity, and making the best clinical decision are among the main ethical challenges of caring for burn patients. It appears that by providing a culturally, professionally, and organizationally suitable environment, where the ethical challenges during caring for burn patients are largely eliminated, administrators can help the caregivers to have more psychological peace and to perform better. The findings of the present study can help health policymakers and administrators in creating a supportive atmosphere free of ethical distress in which quality care can be provided.

## Data Availability

The datasets used and/or analyzed during the current study are available from the corresponding author on reasonable request.
